# An incubation water eDNA method for a non-destructive rapid molecular identification of *Pinna nobilis* and *Pinna rudis* bivalve juveniles

**DOI:** 10.1016/j.mex.2022.101708

**Published:** 2022-04-21

**Authors:** Gaetano Catanese, José Tena-Medialdea, Marija Aleksandra Bel Dajković, Milena Mičić, José Rafaél García-March

**Affiliations:** a Laboratorio de Investigaciones Marinas y Acuicultura (LIMIA) - Govern de les Illes Balears, Av. Gabriel Roca 69, 07157 Port d'Andratx (Balearic Islands) Spain; b INAGEA-UIB, Carr. de Valldemossa, km 7.5, 07122 Palma, (Balearic Islands) Spain; c Instituto de Investigación en Medio Ambiente y Ciencia Marina (IMEDMAR-UCV). Universidad Católica de Valencia. Avenida Puerto Pesquero s/n, 03710 Calpe (Spain); d Aquarium Pula d.o.o., Ulica Verudella 33, 52100 Pula (Croatia)

**Keywords:** Pen shell, eDNA, Molecular biology, Multiplex-PCR

## Abstract

The pen shell *Pinna nobilis* is critically endangered due to a disease that has affected all open water populations since late 2016. Collection of early spats is considered a fundamental step for pen shell conservation. However, the identification between *P. nobilis* and *P. rudis* juveniles by morphology is a very difficult task. Furthermore, due to the small size of juveniles and high sensitivity to handling, the sampling for this purpose must not damage individuals. As a consequence, the application of molecular techniques for conservation strategies to identify threatened and endangered bivalve species is every day more and more necessary.

In this study, we present the development of a multiplex-PCR procedure for the rapid identification of two Pinna species from eDNA water samples. Using species-specific primers, designed in the rRNA16S and rRNA12S mitochondrial genes, identification of species was obtained by cellular or extracellular DNA dissolved in water and differentiated based on the size of the amplified DNA fragments.

• Development of a molecular multiplex-PCR procedure for the rapid identification of two Pinna species from eDNA water samples

• Using specie-specific primers, the different species can be differentiated basing on the size of the amplified DNA fragments

• This technique removes many of the limitations commonly associated with sampling of threatened and endangered juvenile bivalves for conservation strategies (sampling does not damage individuals).

## Graphical abstract

**Figure:**
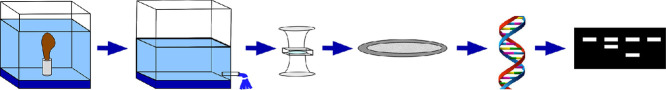

**Specifications table**

**Table:** 

Subject Area:	Environmental Science
More specific subject area:	Molecular Biology
Method name:	eDNA method for Pinna spp. identification
Name and reference of original method:	Not applicable
Resource availability:	Not applicable

## Method details

**Fig. 1:**
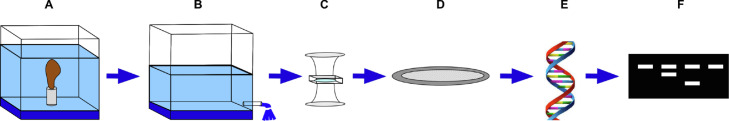
Summary of the method used: not-identified juvenile of Pinna placed in a 200 mL aquarium during 24H (A), later the specimen was removed and the aquarium emptied (B). The water was filtered (C) using a sterile cellulose membrane with diameter 0.45 µm (D). Total DNA was extracted (E) from this filter, employing an appropriate kit. Finally, the purified DNA was applied in a multiplex PCR with specific primers for species identification and the results were visualized in an agarose gel (F).

In this work, we developed a multiplex PCR procedure for the rapid identification of the two Pinna species from water filtered samples in which living juveniles have been housed, thus avoiding damage to the specimens that could be caused during the tissue sampling. Multiplex PCR is a technique based on the simultaneous amplification of more DNA fragments in a single reaction. For this reason, we designed primers in rRNA16S and rRNA12S mitochondrial genes (whose specificity was assured) so that PCR amplicons have a different size in relation to each considered species (Fig. 1).

Selective amplification of a DNA region was achieved by designing primers whose 3′-OH end was located in a species-specific nucleotide polymorphism of the molecular marker. In this way, PCR products could be differentiated in an agarose gel electrophoresis. To make that, it was essential to optimize the conditions of the PCR reaction, such as adjusting the amount of the primers that were added to the reaction for obtaining the specific amplification of each fragment. In addition, the most suitable conditions of temperature and concentration for a high amplification efficiency were established. We tested the PCR protocol using tissue of both pen shell species before using filtered water samples.

### Sampling and DNA extraction

Forty bivalve juveniles of *Pinna* spp., captured in the wild and distinguished from the other mollusks by morphological characteristics were hosted each one during 24 hours in separate aquaria. Water samples (200 ml each) from each aquarium were filtered through a 0.45 µm screen. The dry filters were conserved in Eppendorfs and stored at -80°C. DNA extractions were carried out by DNeasy PowerWater kit (Qiagen), according to the manufacturer's instructions, using a half filter of each sampling (the other half was stored). DNA quality and quantity were tested with a Nanodrop 2000 instrument (ThermoScientific) and used for multiplex-PCR. For samples used as controls, mantle tissues from *P. rudis* and *P. nobilis* adult dead individuals were collected. Total DNA was extracted from mantle using the NucleoSpin® Tissue DNA Isolation Kit (Macherey-Nagel), according to the manufacturer's instructions.

### PCR amplification

For the development of a Pinna-specific procedure applicable to these kinds of samples, only amplicons with a maximum size of 200 bp were considered in the primer design. It is an important issue since DNA could be severely degraded by nuclease enzymatic activities**.**

**Fig. 2:**
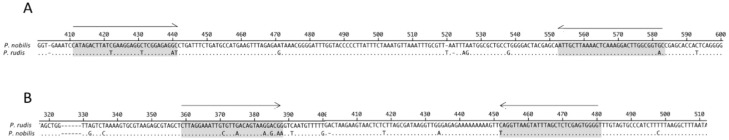
Position of specific primers for amplifications. A) Forward and reverse amplification primers designed on the rRNA12S gene for *P. nobilis* identification; B) Forward and reverse amplification primers designed on the rRNA16S gene for *P. rudis* identification.

In the procedure, specific primers have been designed for each of the two species of the genus *Pinna* (Fig. 2; Table 1). These primers have been designed in such a way that, after multiplex PCR amplification and subsequent visualization of the amplified products on an agarose gel, the different species can be differentiated based on the size of the amplified DNA fragments. The system also included a pair of primers that amplify a DNA fragment of nuclear rRNA28S in both species (and in *Atrina pectinata*), which acts as a positive amplification control. In this way, it can be ruled out that the absence of a specific band is due to PCR inhibition problems

**Table 1:** Primers for multiplex PCR amplifications.

Gene	Primers	Sequence (5’-3’)	Size	TM
rRNA 28S	Pinna28S·F	AAAGGCGCATGAAGTGAAGGCAACCTCG	205 pb	72°
	Pinna28S·R	TTTGCACGTCAGAATCGCTACGGACCT		70°
rRNA 12S	Pnob12S·F	CATAGACTTATCGAAGGAGGCTCGGAGAGGC	175 pb	75°
	Pnob12S·R	GCACCGCCAAGTCCTTTGAGTTTTAAGCAAT		71°
rRNA 16S	Prud16S·F	CTTAGGAAATTGTGTTGACAGTAAGGACGG	123 pb	69°
	Prud16S·R	ACCCCACTCGAGAGCTAAATACTTAACCTG		71°

For the amplification of specific fragments, detailed analyses of the mitochondrial rRNA sequences were first performed.

The regions to search for *Pinna* specific primers for identification were chosen by alignment of those DNA sequences present in our database and in GenBank (access. n. MN432488 and LC634517), Moreover, the following bivalve species mitogenomes, described by other authors and retrieved from Genbank, were used for comparing sequence analysis: *Atrina pectinata* (NC020028), *Crassostrea virginica* (AY905542), *C. gigas* (NC001276), *Ostrea edulis* (JF274008), *Mytilus edulis* (NC006161) and *M. galloprovincialis* (NC006886).

Once the mitochondrial ribosomal genes aligned with Pinna and other bivalve species, the suitability and a clear nucleotide differentiation among each species was demonstrated. Then species-specific primers for both Pinna nobilis and P. rudis were designed.

For rRNA12S, a total of 59 polymorphic sites were detected in the alignment of all haplotypes. The inter-specific mismatches were evaluated as possible diagnostic positions. Thus, the positions 393–423 of the rRNA 12S gene were chosen as optimal to design the forward primer (Pnob12sF) because the sequence clearly permitted observation of the P. nobilis-specific nucleotide differences at the critical 3-end (GC in *P. nobilis*, AT in *P. rudis*; Fig. 2A). This *P. nobilis*-specific sequence stretch was completely conserved in all the haplotypes studied in this work. Moreover, a non-selective reverse primer (Pnob12sR) was situated between positions 534–564 (Fig. 2A), exhibiting appropriate thermodynamic characteristics for pairing with forward primers. The amplicon expected was 175 bp in length. In addition, more than 10 nucleotide differences were observed in the comparison of the sequence of the primer regions in the other bivalves used in this study, including the taxonomic nearest *A. pectinata*.

Similarly, 91 nucleotide differences were observed between the complete rRNA 16S gene sequences of the two Pinna species. The positions 353–382 of alignment were chosen as optimal for *P. rudis*-specific forward primer (Prud16SF), which showed nucleotide differences at the critical 3-end (GG in *P. rudis*, AA in *P. nobilis*, Fig. 2B). The other selective reverse primer (Prudis16R) was situated between positions 445–474 with only the critical 3-end in the position 445 (C in *P. rudis*, T in *P. nobilis*; Fig. 2B). The other bivalves also showed greater than 8 differences in the sequence of the region of primers. The expected length of this specific amplicon was 123 bp.

Therefore, this procedure was based on the use of three pairs of primers:

I - Positive amplification control. Primers Bivalve28S•F and Bivalve28S•R designed in highly conserved regions of the 28S rRNA nuclear ribosomal gene are used. These primers generate a 205 bp amplicon. It allows to rule out the existence of inhibitor compounds of the PCR reaction.
II - Identifying fragment of *P. nobilis*. Primers Pnob12S•F and Pnob12S•R designed from specific sequences of the mitochondrial ribosomal gene 12S rRNA were used. These primers generate a 175 bp amplicon.
III - Identifying fragment of *P. rudis*. Primers Prud16S•F and Prud16S•R designed from specific sequences of the mitochondrial ribosomal gene 16S rRNA are used. These primers generate a 123 bp amplicon.

The multiplex PCR reaction using DNA was carried out in a total volume of 20 µL (microliters) containing: 10 µL (1.25 units) of KAPATaq DNA polymerase (Merck), 0.15 µL of each primer Bivalve28S•F and Bivalve28S•R (20 µM), 0.45 µL of each primer Pnob12S•F and Pnob12S•R (20 µM) (specific for *P. nobilis*), 0.45 µL of each primer Prud16S•F and Prud16S•R (20 µM) (specific for *P. rudis*), 1 µL of template DNA from the sample (∼ 2–15 ng) and sterile distilled water until the total volume of 20 µL.

The PCR amplifications were performed with KapaTaq DNA polymerase under the following conditions: an initial denaturation step at 95 °C for 2 min, followed by 38 cycles of denaturation at 95 °C for 30 s, annealing at 64 °C for 10 s and elongation at 72 °C for 20 s.

The PCR products were loaded on high-resolution agarose gels, type Ultrapure ™ Agarose 1000 (Invitrogen), at a percentage no less than 2.5%, and stained with GelRed (Biotium). Electrophoresis was carried out using 1x TBE buffer. Detection of DNA fragments was done by visualization with ultraviolet light. The standard 50 bp DNA Ladder (Norgen) was used as a standard to determine the size of DNA fragments.

The method allows the direct identification of each *Pinna* species simply by the observation of different PCR band sizes in agarose gel and without requiring further sequencing, as described in the next paragraph (Validation of method).

## Validation of the method

**Fig. 3:**
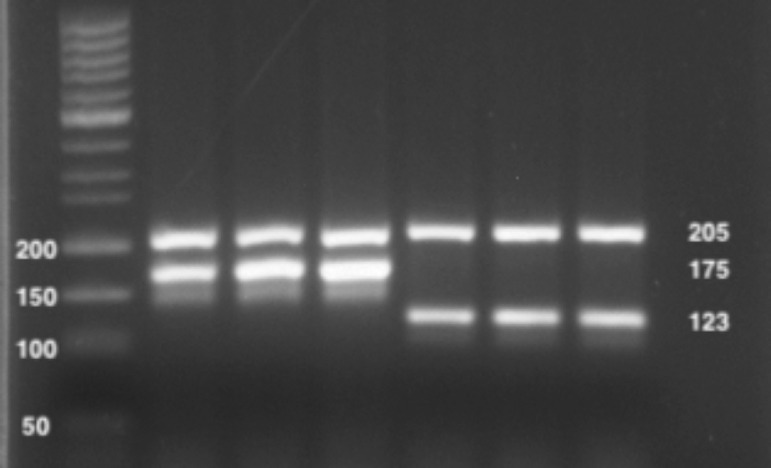
Electrophoresis in Agarose gel of multiplex-PCR products from DNA of *Pinna* spp. Band sizes of the 50 bp DNA Ladder are indicated on the left. Sizes of the generated products are shown on the right. Lanes 1: containing the 50 bp DNA ladder; Lanes 2–4: *P. nobilis* samples; Lanes 5–7: *P. rudis* samples

As expected, using these highly restrictive PCR conditions, all samples showed the PCR product corresponding to the 205 bp fragment amplified from the 28S rRNA gene from the two species (which shows that the PCR reaction worked well), and one of the two diagnostic fragments: the 175 bp PCR product derived from the 12S gene from *P. nobilis*, or the 123 bp PCR product amplified from the 16S gene from *P. rudis* (Fig. 3). No cross-amplification of the specific fragments was observed. The method was also validated using mantle tissue from dead adult individuals. Additionally, the specificity of the multiplex test was assayed in samples of other 4 bivalves (a total of 8) such as *A. pectinata, M. edulis, M. galloprovincialis* and *O. edulis*, using the same PCR conditions.

Moreover, in samples giving a positive result with *P. nobilis*- and *P. rudis*-specific primer pairs, identifications were confirmed by purifying and sequencing the amplicon generated with the primers designed for verification.

### Sequencing for species identification to check the test

To ensure the correct identification of species, all water samples were also subjected to PCR and sequencing for the 12S and 16S rRNA gene larger fragments.

Two new primer pairs were designed for sequencing partial rRNA genes of *Pinna* spp. in water samples and mantle of adults. The primers Bivalve12sF (5’- ACGCTGGTTTAAATTCACAACTCAAGT-3’) and Bivalve12sR (5’- CTACCGTGTTACGACTTACCTCCACTTT-3’), Bivalve16sF (5’-TACAGGTGAATTCTGGTACCTTTTGC-3’) and Bivalve16sR (5’-GAACTGGCTTACGCCGTTCTGAACT -3’) were used for amplifying and sequencing approx. 700 bp and 1.1 kb of the rRNA12s and rRNA16S genes respectively, for identifying *P. nobilis* and *P. rudis* species in each sample. PCR reactions were carried out in a final volume of 20 µL containing: 10 µL (1.25 units) of KAPATaq DNA polymerase (Merck), 0.4 µL of each primer 1 µL of template DNA from the sample (∼ 10 ng) and sterile distilled water, and performed at the following conditions: 94°C for 2 min; 40 cycles of 30 s at 9 °C, 20 s at 54 °C and 1.10 min at 72 °C. PCR products were purified using a QIAquick PCR purification kit (Qiagen) and bidirectionally sequenced using the Sanger's method with a 3730XL DNA Analyzer (Applied Biosystems).The nucleotide sequences of *P. rudis* and P. nobilis were individually aligned with MEGA X software.

This application of sequencing to samples, were compared with the reference sequences of the mitogenome of *P. nobilis* (GenBank access. n. MN432488) and *P. rudis* (GenBank access. n. LC634517), confirmed positive and correct identifications in all respective cases.

## Supplementary material *and/or* Additional information

### Background

The congeneric pen shells *P. nobilis* and *P. rudis* are large wedge-shaped bivalve mollusks, belonging to the family Pinnidae. *P. rudis* is widely distributed in the world's oceans, but *P. nobilis* only inhabits Mediterranean Sea [1]. As benthic, sessile filter-feeders, they play an important role in ecosystems [2]. *P. nobilis* populations have undergone a significant reduction in individual number during the last few years, due to a mass mortality event (MME) [3]. The species is now included as “critically endangered” in the IUCN Red List [4] and as endangered with extinction in Spanish coasts (Orden TEC/596/2019, de 8 de abril). The MME has been mainly associated to the presence of pathogens such as: the parasite *Haplosporidium pinnae* [5], [6], [7], *Mycobacterium sp*. [8,9], *Vibrio spp*. [10,11] or mixing of them. However, recently it has been proven that the infection by *H. pinnae* plays a main role on the onset of the mass mortality of *P. nobilis* throughout the Mediterranean Sea [6]. Curiously, *P. rudis* seems not to be affected and events of mortality caused by pathogens have not been described in this species until now [5,6].

The reduction of the populations of *P. nobilis* has led nowadays to the existence of only a few areas with relic populations, located in coastal lagoons and deltas [6,[12], [13], [14], [15]]. Among the many measures that national authorities and researchers are trying to implement for the safeguarding of individuals and the recovery of populations [12,16], is an international network of larvae collectors deployed in summer months, which has been growing since the onset of the MME [17]. These collectors serve as a fixing point for pelagic larvae, which find an attractive habitat for their settlement while swimming in the water column. On the other hand, the settlement of larvae on the ropes of aquaculture facilities on the Spanish coast has also been very remarkable, with thousands of *Pinna* spp. individuals recruited in a single season [18]. *Pinna* spp. juveniles grow within the collectors or in the ropes up to 2–3 cm, until retrieved for their study in laboratory [17,18]. Although it is possible to differentiate *Pinna* spp. from other mollusks, due to their small size it is impossible to distinguish between the two Mediterranean *Pinna* spp. and, therefore, it is necessary to maintain them in tanks, until they grow and develop the specific shell features. This is inconvenient, both because more than six months might be necessary for the juveniles to express their shell characteristics in the tanks (García-March pers. obs.) and because all individuals have to be treated as if they were *P. nobilis* juveniles infected by *H. pinnae*. Another extra inconvenience of maintaining the juveniles in the tanks, without identifying between the two species, is that some of them die during this period, before displaying any distinctive shell feature. For these deceased juveniles, researchers can only suppose they could be infected *P. nobilis*. They are so small that usually, when found dead, they are already empty of soft tissues, impeding their genetic identification. Additionally, the consideration of all juveniles as infected *P. nobilis*, requires specific food supplementation [18], extra water filtering to 0.5 µm, and cold temperatures <14 ºC, which are not appropriate for *P. rudis* growth [19], which further delays their identification. On this regard, it would be much better to separate both species and put all *P. rudis* in suspended cages in the water column, where they can grow more than 3 cm/month [19] and maintain only the *P. nobilis* juveniles in the tanks [16]. Note that, until now, all *P. nobilis* juveniles recruited in situ die due to the parasite [17] and hence, maintaining them in situ, within anti-predator cages in the water column, is inadvisable. Additionally, one of the reasons of installing larvae collectors is identifying the areas of concentration of *P. nobilis* larvae and evaluating their potential source [17]. This is impossible if both species cannot be separated. By this reasoning, the development of a rapid method to identify both species just after they have been retrieved, would improve considerably the selection of the juveniles and would be a fundamental tool for the recovery of *P. nobilis*. Also, it would help to identify the areas were more *P. nobilis* juveniles are being collected to increase the effort of collector installation in these areas and evaluate the source of larvae.

During the last years, the application of Environmental DNA (eDNA) methods is increasingly being used for the identification of organisms through analysis of DNA that can be found in the environment. Environmental DNA originates from cellular material, including nuclear or mitochondrial DNA, shed by organisms (via skin, excrement, etc.) into aquatic environments that can be sampled and monitored using molecular methods [20]. Sources of eDNA including secreted feces, mucous, and gametes as well as shed skin and fish scales or carcasses can be detected in cellular or extracellular (dissolved DNA) form. Recently, this technology has been successfully employed for the detection of five highly invasive mollusks in urban rivers [21]. At the same time, new genetic works have been providing new insights about the mitochondrial genomes and nuclear markers of Pen shells [22], [23], [24]. Additionally, putative hybrids, showing shell morphology and mantle coloration intermingled exhibiting both *P. nobilis* and *P. rudis* traits and diagnostic nucleotides in 28S rRNA gene between the two species, were also recently described [25].

## Declaration of Competing Interest

The authors certify that the material presented here is original work which has not been previously published. All biological samples were collected during management operations as part of a study plan approved by the Generalitat Valenciana (Conselleria de Agricultura, Medio Ambiente, Cambio Climático y Desarrollo Rural) and the Spanish Ministry for the Ecological Transition and the Demographic Challenge. Appropriate ethics, permissions to manipulate animals and other approvals were obtained for the submitted research.

X The authors declare that they have no known competing financial interests or personal relationships that could have appeared to influence the work reported in this paper.
